# Novel compound heterozygous mutation in the *CNGA1* gene underlie autosomal recessive retinitis pigmentosa in a Chinese family

**DOI:** 10.1042/BSR20150131

**Published:** 2016-01-22

**Authors:** Xin Jin, Ling-Hui Qu, Bao-Ke Hou, Hai-Wei Xu, Xiao-Hong Meng, Chi-Pui Pang, Zheng-Qin Yin

**Affiliations:** * Southwest Hospital, Southwest Eye Hospital, Third Military Medical University, Chongqing 400038, China; † Department of Ophthalmology, General Hospital of Chinese PLA, Beijing 100853, China; ‡ Key Lab of Visual Damage and Regeneration & Restoration of Chongqing, Chongqing 400038, China; § Department of Ophthalmology and Visual Sciences, Faculty of Medicine, The Chinese University of Hong Kong, Hong Kong SAR 999077, China

**Keywords:** *CNGA1*, mutation, next-generation sequencing, retinitis pigmentosa

## Abstract

Retinitis pigmentosa (RP) describes a group of inherited retinopathies that are characterized by the progressive degeneration of photoreceptor neurons, which causes night blindness, a reduction in the peripheral visual field and decreased visual acuity. More than 50 RP-related genes have been identified. In the present study, we analysed a Chinese family with autosomal recessive RP. We identified a compound heterozygous mutation, c.265delC and c.1537G>A, in *CNGA1* using targeted next-generation sequencing (NGS) of RP-causing genes. The mutations were validated in the family members by Sanger sequencing. The mutations co-segregated with the RP phenotype and were absent from ethnically-matched control chromosomes. The mutant (mut) CNGA1 p.(G513R) protein caused by the mis-sense novel mutation c.1537G>A was expressed *in vitro*. The mut CNGA1 p.(G513R) protein was largely retained inside the cell rather than being targeted to the plasma membrane, suggesting the absence of cGMP-gated cation channels in the plasma membrane would be deleterious to rod photoreceptors, leading lead to RP.

## Abstract (precis)

A novel compound mutation in *CNGA1* gene, coding for the cGMP-gated ion channel protein, results in a protein product that is not targeted to the plasma membrane, which would be deleterious to rod photoreceptors leading to retinitis pigmentosa (RP).

## INTRODUCTION

Retinitis pigmentosa (RP; OMIM 268000) is a group of inherited retinopathies that are characterized by the progressive degeneration of photoreceptor neurons, resulting in night blindness, a reduction in the peripheral visual field and decreased visual acuity. The estimated prevalence of RP is ∼1:3000–1:5000 [1,2]. RP is generally transmitted via autosomal dominant (ADRP), autosomal recessive (ARRP) and X-linked (XLRP) inheritance. However, digenic or mitochondrial methods of inheritance and occasional pedigrees that display so-called digenic-diallelic or digenic-triallelic hereditary patterns have also been reported [3]. To date, mutations in 57 genes have been associated with RP in the Retinal Information Network (RetNet: http://www.sph.uth.tmc.edu/retnet/sum-dis.htm, search performed August 24, 2015), hence making genetic analysis extremely non-feasible. Hence, given the genetic heterogeneity of RP and the need to be able to make an accurate and comprehensive molecular diagnosis it is imperative to have complete knowledge about the pathogenic gene spectrum in RP. Such knowledge will also aid confirmation of clinical diagnosis and assessment of long-term disease prognosis as the basis for novel therapeutic approaches and personalized medicine.

*CNGA1* (*CNGA1*; OMIM 123825) encodes the α-subunit of the rod cyclic cGMP-gated cation channel (CNG), which is a non-selective ion channel protein that is involved in the final stage of the phototransduction pathway [4]. The retinal rod cGMP-gated cation channel is a hetero-oligomer composed of two homologous subunits, α (CNCG1) and β (CNCG2), each of which consists of a core structural unit of six membrane-spanning segments, a pore region and a cGMP-binding domain [5,6]. The α-peptide forms a functional channel alone and is considered to be the main functional subunit [7]. Mutations within the *CNGA1* gene cause the RP49 form that presents with an early onset and severe retinal degeneration. Studies suggest *CNGA1* mutations are rare in European RP patients in comparison with Asian RP patients [8–10]. The prevalence of *CNGA1* mutations in Chinese and Japanese populations with ARRP were 7.6% and 5.1% compared with 2% in a Spanish population. [8–10].

In the present study, we identified the compound heterozygous *CNGA1* mutation c.265delC and c.1537G>A in a Chinese ARRP family. We used a combined approach of targeted next-generation sequencing (NGS) and candidate mutation validation to identify the causative mutations. Next, we generated expression constructs for GFP-tagged wild-type (wt) and mutant (mut)-CNGA1 proteins c.1537G>A p.(G513R) and expressed them in human embryonic kidney cells (HEK)-293 to determine if and how the c.1537G>A mutation affected the normal expression of CNGA1. Our findings demonstrated association between the c.1744G>A mutation and ARRP and thus expands the pathogenic genetic spectrum of *CNGA1* in RP.

## MATERIALS AND METHODS

### Pedigree and controls

The present study followed the tenets of the Declaration of Helsinki and received approval from the Ethics Committee of Southwest Hospital. Signed informed consent was obtained from all study participants.

**Figure 1:**
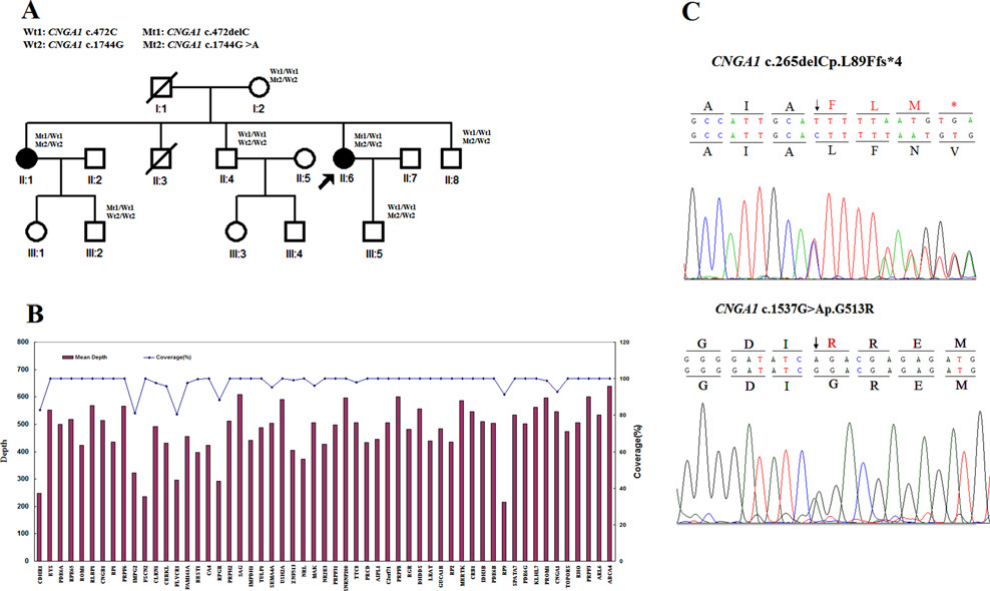
Identification of the compound heterozygous mutation c.265delC and c.1537G>A in *CNGA1* in a Chinese family with ARRP (**A**) Family pedigree. Squares and circles indicate males and females respectively. Darkened symbols represent the affected individuals and slashed symbols denote that the subject is deceased. The patient indicated by the arrow is the proband. (**B**) Summary of the 55 RP-causing genes contained in the NGS capture array. (**C**) The heterozygous *CNGA1* mutations identified by Sanger sequencing. The arrows indicate the site of the mutations.

A three-generation ARRP pedigree (Figure 1A) from Sichuan province, China, was analysed and followed up clinically at the Southwest Eye Hospital. There were 15 members in the ARRP family and 13 individuals were recruited for the study. Among these, two were affected individuals and 11 were unaffected, of which three were unrelated spouses. Normal controls were enrolled from the genetic resources database of Southwest Eye Hospital, including 500 unrelated individuals aged >50 years who had no symptoms of night blindness, vision reduction or any personal or family history of known inherited diseases. Comprehensive ophthalmological examinations, including best correct visual acuity, applanation tonometry, fundus examination, fundus autofluorescence (FAF), spectral-domain optical coherence tomography (SD–OCT), electroretinogram (ERG) and multifocal ERG (mfERG) were performed in both affected individuals (II: 1 and II: 6, Figure 1A). Other members in the family with no symptoms of RP received the fundus examination to exclude RP diagnosis. ERGs were performed and recorded according to the standard testing protocols for clinical electroretinography (ISCEV13) and the guidelines recommended by the International Society of Electrophysiology of Vision for basic mfERG [11]. Genomic DNA was prepared from peripheral blood lymphocytes of the pedigree members and normal controls using a QIAamp DNA Blood Midi Kit (Qiagen).

### Targeted region capture and next-generation sequencing

A custom-made capture array (Roche NimbleGen) designed by the Beijing Genomics Institute (BGI), was used to capture the exons coding 55 RP-related genes (Figure 1B). Genomic DNA from proband II:6 (Figure 1A) was pooled and hybridized to the custom capture array for 72 h at 42°C. After hybridization, the array was washed and eluted according to the manufacturer's instructions (Roche NimbleGen). The captured library was sequenced using an Illumina HiSeq2000 (Illumina) analyser for 90 cycles per read to generate paired-end reads according to standard sequencing protocols. Image analysis and base calling were performed using the Illumina Pipeline to generate raw data.

### Variant identification and validation

To detect the potential pathogenic variants of the proband, we aligned the sequencing reads against the human genome reference information in the National Center for Biotechnology Information (NCBI) database (version hg19) using the Burrows Wheeler Aligner Multi-Vision software package. Single-nt variants (SNVs) and indels were identified using SOAPsnp software and Samtools Indel Genotyper respectively [12,13]. The SNVs were included in analysis if they met all of the following criteria: (i) quality score ≥20 (Q20); (ii) average copy number at the allelic site ≤2; (iii) distance of two adjacent single-nt polymorphisms (SNPs) ≥5 bp; and (iv) sequencing depth ≥4 and ≤500. For analysing indels, Burrows–Wheeler Aligner (BWA) was used to map reads on to the reference. The mapped reads were passed to GATK release 1.5 software (http://www.broadinstitute.org/gsa/wiki/index.php/Home_Page) to identify the breakpoints and to annotate the genotypes of insertions and deletions. All SNVs and indels were compared with several databases including NCBI dbSNP (http://hgdownload.cse.ucsc.edu/goldenPath/hg19/database/snp132.txt.gz.), HapMap project (ftp://ftp.ncbi.nlm.nih.gov/hapmap), 1000 Genomes Project (ftp://ftp.1000genomes.ebi.ac.uk/vol1/ftp) and a database of Chinese healthy adults from BGI. Common variants were removed and the variants with previously known ARRP relevance were retained for further analysis with variants present in a homozygous state and compound heterozygous were retained for recessive inheritance modes of this pedigree. The remaining variations of known RP causative genes were validated by PCR and Sanger sequencing to determine whether the variations segregated with the RP phenotype. PCR primer sets were designed using Primer6.0 (Table 1); the products were sequenced using a Bigdye terminator v3.1 cycle sequencing kit (Applied Biosystems) and analysed on an ABI 3730XL Genetic Analyzer.

**Table 1:** Primers used for potential pathogenic mutations amplification

Mutation	Gene	Exon	Forward primer (5^′^-3^′^)	Reverse primer (5^′^-3^′^)	Product length (bp)
c.265delC	*CNGA1*	6	tgagtagaaatggagaagaatttgttt	tgaacactacttttcaacaaaatga	329
c.1537G>A	*CNGA1*	11	tgctgattgtgaagctggtc	cgattgccagctttgctc	240

### Expression of the mutated alleles *in vitro*

cDNA encoding *CNGA1* was inserted into the pIRES2–EGFP eukaryotic expression vector (Clontech), which contains the internal ribosome entry site of encephalomyocarditis virus between the multiple cloning site and the EGFP coding region. Wt *CNGA1* cDNA was mutated *in vitro* to produce the p.(G582R) variant with the QuickChange® Site-Directed Mutagenesis Kit (Stratagene). To express the proteins, HEK-293 cells were cultured in Dulbecco's modified Eagle medium (DMEM; Thermo Scientific HyClone) supplemented with 10% FBS (Thermo Scientific HyClone) in 5% CO_2_ and 95% air at 37°C. Cells were transfected at 70%–90% confluence using TurboFect™ *in vitro* Transfection Reagent (Thermo Fisher Scientific Fermentas) according to manufacturer protocol. Briefly, 1 μg of cDNA was diluted with 100 μl of serum-free DMEM followed by 2 μl of Turbofect transfection reagent. After a 15-min incubation at room temperature, the transfection reagent mixture was applied to the cells, which were then cultured for an additional 48 h before use. Transfected cells were identified using fluorescence microscopy and selected for further analysis.

### Immunocytochemistry

Transfected HEK-293 cells were harvested, centrifuged, resuspended and pipetted on to poly (D-lysine)-coated microscope slides. The cells were fixed in 4% paraformaldehyde in 0.1 M PBS for 30 min at room temperature, rinsed in PBS and then blocked with 10% goat serum and 0.1% Triton X-100 (Sigma–Aldrich) for 1 h. The cells were then incubated with antibodies against CNGA1 (1:500; AbCam) overnight at 4°C followed by 1 h at 37°C. After three washes in PBS, the cells were subsequently incubated with Cy3-conjugated secondary antibodies (1:100; Santa Cruz Biotechnology) for 1 h at 37°C. The slides were then rinsed with PBS, mounted in anti-fade mounting agent with DAPI (Beyotime Institute of Biotechnology) and observed using a fluorescence microscope (Olympus BX51).

### Western blotting

Wt- and mut-CNGA1 proteins were isolated from transfected HEK-293 cells using NP-40 lysis buffer (Biyuntian). Twenty micrograms of protein from each sample were resolved by SDS/PAGE (Amresco) and transferred to PVDF membranes (Millipore). The membranes were probed with primary antibodies against β-actin (loading control, 1:1000; Santa Cruz Biotechnology) and CNGA1 (1:500, AbCam).

## RESULTS

### Clinical findings

**Figure 2:**
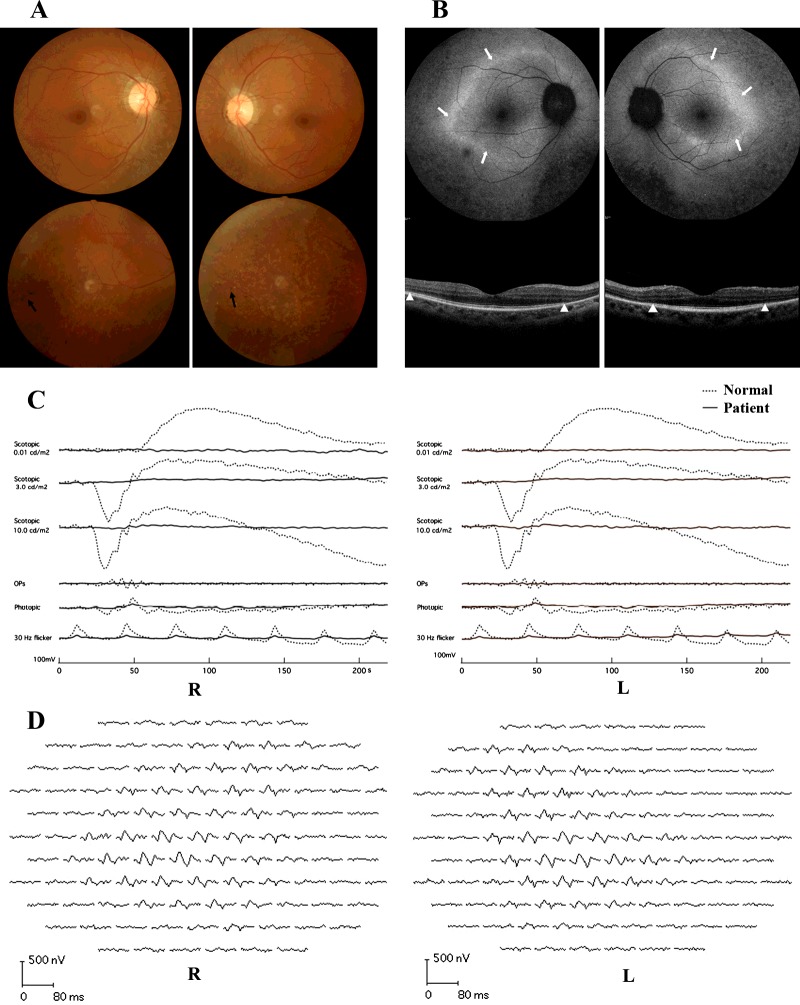
Ophthalmological examination of the proband (**A**) Bilateral fundus image showing attenuation of the retinal arterioles, atrophy of the retinal pigment epithelium and a waxy-pale disc. A small amount of pigmentation was found in the periphery of the retina, as indicated by the white arrow. (**B**) An abnormal high density ring in FAF, marked by the white arrows and a discontinuous photoreceptor IS/OS junction line in SD–OCT, indicated by the white triangle, was observed. (**C**) Undetectable ERG responses were observed in dark-adapted (DA) conditions at 0.01 Hz, DA 3.0 Hz and DA 10.0 Hz, whereas significantly reduced responses were observed in light-adapted (LA) conditions at 3.0 Hz and LA 30 Hz. The solid lines represent responses of the proband and dotted lines illustrate responses of the normal control. (**D**) The mfERG traces of the proband demonstrated responses of central segment were preserved, whereas peripheral responses were decreased significantly.

The pedigree of the three-generation Chinese family was consistent with autosomal recessive inheritance (Figure 1A). The two patients presented with similar symptoms: obvious night blindness and a narrowing of the visual field occurred at ∼2 years old and a gradual decline in visual acuity was detected after the age of 40 years. The patients' visual fields were constricted to 10°–15° from the centre in both eyes and visual acuity was 0.8–0.9 (Table 2). A fundus examination showed the appearance of RP with pale discs, arteriolar constriction, retinal pigmented epithelium atrophy and sporadic bone spicule pigmentation affecting the peripheral retina (Figure 2A). FAF imaging revealed an abnormally high-density parafoveal ring (Figure 2B). SD–OCT demonstrated discontinuous photoreceptor inner/outer segment (IS/OS) junction line, the external limiting membrane (ELM) and cone outer segment tips (COST) line in macular (Figure 2B), similar to normal macular structures. A full-field scotopic rod ERG showed extinguished responses and a cone ERG revealed significantly reduced responses (Figure 2C). The mfERGs demonstrated responses of central segment were preserved, whereas peripheral responses were decreased significantly (Figure 2D).

**Table 2:** Clinical and ophthalmological findings in the two affected sibs of the family Abbreviations: BCVA, best corrected visual acuity; F, female; LE, left eye; N, normal; RE, right eye.

Patient	II:1	II:6
Age (years)/Sex	50/F	42/F
Onset (years)		
Night blindness	2–3	2–3
Visual field constriction	6–7	6–7
Decreased visual acuity	40	40
Ophthalmic examination		
BCVA	RE: 0.6/ LE: 0.7	RE: 0.8/ LE: 0.9
Anterior segment	N	N
Fundus	RE/LE : pale disc, artery attenuation, perivascular bone spicule pigment deposits	RE/LE: pale disc, artery attenuation, perivascular bone spicule pigment deposits
Visual field	RE: 15°	RE: 20°
	LE: 15°	LE: 20°
ERG	extinguished	extinguished

### Identification of ARRP-causing mutations

Approximately 21.65 kb of the exons and adjacent intronic regions of 55 RP genes were captured and sequenced from the proband. The mean coverage of the 55 RP genes was ∼98.1% and the median depth was 479× (Figure 1B). A mean 100% of the base pairs with N200x coverage were detected successfully for each gene, indicating a high capability for variant identification.

The *CNGA1* sequence was compared with the NCBI reference sequence for *CNGA1* transcript variant 2 (GenBank ID; NM_000087.3) for allowing direct comparison with known *CNGA1* mutations in The Human Gene Mutation Database, HUGO (http://www.hgmd.cf.ac.uk/ac/all.php). In the proband, 561 SNPs and 122 indels were identified in the coding regions and introns that might affect splicing. After filtering the candidate variants in the databases, two heterozygous SNVs in the coding region of the *CNGA1* gene were identified: c.265delC in exon 6 and c.1537G>A in exon 11 (GenBank ID NM_00087.3). At the translational level, the c.265delC mutation was predicted to result in a frameshift that caused a premature termination codon p.(L89Ffs*4), whereas c.1537G>A was predicted to cause an amino acid substitution of arginine for glycine at position 513 p.(G513R). The polyphen-2 and SIFT program predicted p.(G513R) could cause severe damage to the protein.

To confirm the *CNGA1* variants and assess the inheritance pattern of RP in the proband, Sanger sequencing was used to analyse the variants in the proband and her family members (Figure 1C). Both affected individuals carried this compound heterozygous mutation, whereas the unaffected members carried single heterozygous mut or wt sequence, i.e. the mutations co-segregated with the RP phenotype in the family and were absent from the normal control database (Figure 1A).

### Expression of the mutant alleles *in vitro*

**Figure 3:**
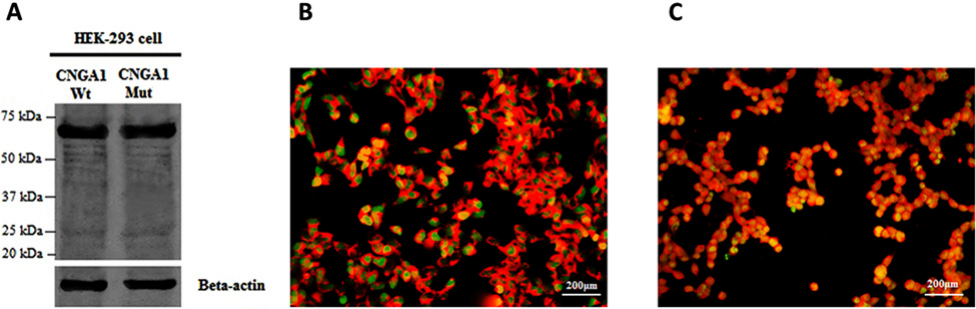
Evidence for mistargeting of the mis-sense mutated protein p.(G513R) *in vitro* (**A**) Results of western blotting (WB). Wt- and mut-CNGA1 were abundantly expressed in HEK-293T cells and could be detected by anti-CNG1 antibodies. Anti-β-actin antibodies were used as a loading control. (**B** and **C**) Immunofluorescence staining using anti-CNGA1 antibodies in transfected HEK-239 cells. (**B**) Wt-CNGA1 protein (red) was expressed in the plasma membrane and EGFP (green) was expressed in the cytoplasm. (**B**) Mut-CNGA1 p.(G513R) was localized in the cytoplasm and was co-expressed with EGFP (yellow). Scale bar=200 μm.

To investigate if and how the p.(G513R) mut affected CNGA1 protein expression and function in RP pathogenesis, we ectopically generated and expressed wt- and mut-*CNGA1* plasmids and expressed them in HEK-293 cells. Both wt- and mut-CNGA1 were abundantly expressed and there was no observable difference in their steady state expression (Figure 3A). However, whereas the wt-CNGA1 protein expression was largely localized to the plasma membrane (Figure 3B), the mut-CNGA1 p.(G513R) protein localization was segregated to the cytoplasm (Figure 3C). Cumulatively, our data indicate that the c.1537G>A mutation prevents the CNGA1 protein from being transported to the membrane, where it is functional in a normal eye. The mechanism(s) underlying the failure of the mut CNGA1 protein to translocate to the plasma membrane remains to be investigated.

## DISCUSSION

RP describes a group of heterogeneous genetic diseases that are caused mainly by pathogenic genes. However, the identification of RP-causing mutations using traditional methods is challenging because of the large number of disease-causing genes. NGS technology provides a high-throughput and cost-effective method for the molecular diagnosis of RP [14–17]. The causative mutations of ∼50%–80% RP cases could be detected successfully using this method and the percentage of novel pathogenic alleles is approximately 45%–60% [18,19]. In the present study, we identified a compound heterozygous mutation c.265delC and c.1537G>A in *CNGA1* in a Chinese family with ARRP using a combined approach of NGS sequencing and candidate mutation validation. The compound heterozygous mutation co-segregated with the RP phenotype in the pedigree and were absent from normal control databases. We also present evidence to suggest that the mis-sense allele *CNGA1* c.1537G>A leads to a mistargeted protein product that does not localize to the plasma membrane where it normally functions in rod photoreceptors.

Visual phototransduction relies on the function of CNG in the photoreceptor outer segment plasma membranes. In the dark, the CNG channel is maintained in the open state by a high concentration of cGMP produced by retinal guanylate cyclases, which results in depolarization of the photoreceptor and synaptic glutamate release. When visual pigments absorb light, G protein (transducin)-mediated signalling activation of retinal cGMP phosphodiesterase leads to hydrolysis of cGMP and closure of the CNG channel. As a result, the photoreceptor hyperpolarizes and shuts off synaptic glutamate release. *CNGA1* encodes the human retinal A subunit of the rod channel CNG and plays a very important role in maintaining the normal structure and function of rod photoreceptor cells [20].

There have been previous reports of *CNGA1* mutations associated with ARRP [9,21]. To date, 19 mutations have been identified (http://www.hgmd.cf.ac.uk/ac/gene.php?gene=CNGA1, assessed on August 24, 2015), inclusive of 13 mis-sense/non-sense and six small deletion mutations. Interestingly, the HGMD Public user site lists only eight *CNGA1* mutations and one needs HGMD Professional Access (which we did not have) to view all the 19 *CNGA1* mutations. Of the known mutations, there are three non-sense, one mis-sense and four frameshift mutations [4,8–10,21].

The ARRP family, in the present study, carried compound heterozygous *CNGA1* mutation c.265delC and c.1537G>A, which resulted in a phenotype of classical RP including night blindness, peripheral visual field loss, attenuated vessels, peripheral bone spicule pigmentation, waxy pallor of the optic disc and peripheral chorioretinal atrophy. The phenotypic characteristics of night blindness and contraction of the visual field occurred at a very young age (∼2 years). However, the macular had a relatively normal structure in OCT images and an AF examination, the central segment responses of mfERG were preserved and the patients still had better central vision acuity at the later stage of the disease. The mfERG is a sensitive and objective method to evaluate the preserved central retinal function of RP patients. The amplitude of the central segment of the mfERGs were correlated well with visual acuity [22]. These clinical data suggest that early onset severe night blindness, slow aggravation and retention of macular structure and partial function in later disease might be the characteristic phenotype of this Chinese ARRP pedigree. Although the *CNGA1* mutations are represented in ARRP, there have been few reported cases. It seems that Asian RP patients carry *CNGA1* mutations more frequently than European patients. Considering the phenotypes related to *CNGA1* mutations, retina degeneration in the macular region differed from previous reports. In addition to the typical phenotypes of RP including night blindness, visual field loss, retinal degeneration with pigmentation and attenuation of retinal vessels, some patients experience macular degeneration and severely decreased BCVA, whereas other patients experience only mild damage to macular function at the later stages of disease. The genotype–phenotype correlation for *CNGA1* mutations remains unclear.

In the compound heterozygous *CNGA1* mutation of c.265delC and c.1537>A identified in the present study, the c.265delC mutation has been reported previously in a Chinese and three Japanese RP patients [8,10], therefore, suggesting the founder is specific to Asian populations. Our data seem to support this hypothesis. The mis-sense mutation c.1537G>A has been described as a polymorphism or rare variant, although its pathogenicity was unclear.

Finally, the protein containing the p.(G513R) mutation encoded the full-length protein, but failed to localize to the plasma membrane. These observations strongly indicate that the c.1537G>A mutation leads to mistargeted protein products and suggest the pathogenesis of photoreceptor degeneration in the current pedigree might be the same as other known RP-causing *CNGA1* mutations, which result from the paucity or lack of cGMP-gated channels in the plasma membrane of rod outer segments [4].

In conclusion, we identified two heterozygous *CNGA1* mutations, c.265delC and c.1537G>A, which were associated with ARRP in a Chinese family. The very early onset of severe night blindness and relatively late onset of mild function loss in central retinal were the characteristic phenotypes of these mutations. Experiments *in vitro* suggested that the mis-sense mutation of p.(G513R) prevented protein expression in the plasma membrane, thus indicating its potential role in RP pathogenesis.

## Abbreviations

ARRP: autosomal recessive retinitis pigmentosa
DMEM: Dulbecco's modified Eagle medium
ERG: electroretinogram
FAF: fundus autofluorescence
HEK: human embryonic kidney cells
IS: inner segment
mfERG: multifocal ERG
mut: mutant
NGS: next-generation sequencing
OS: outer segment
RP: retinitis pigmentosa
SD-OCT: spectral-domain optical coherence tomography
SNP: single-nt polymorphism
SNV: single-nt variant
wt: wild-type

## References

[1] Hamel C. (2006). Retinitis pigmentosa. Orphanet. J. Rare. Dis..

[2] Hartong D.T., Berson E.L., Dryja T.P. (2006). Retinitis pigmentosa. Lancet.

[3] Daiger S.P., Bowne S.J., Sullivan L.S. (2007). Perspective on genes and mutations causing retinitis pigmentosa. Arch. Ophthalmol..

[4] Dryja T.P., Finn J.T., Peng Y.W., McGee T.L., Berson E.L., Yau K.W. (1995). Mutations in the gene encoding the alpha subunit of the rod cGMP-gated channel in autosomal recessive retinitis pigmentosa. Proc. Natl. Acad. Sci. U.S.A..

[5] Dhallan R.S., Macke J.P., Eddy R.L., Shows T.B., Reed R.R., Yau K.W., Nathans J. (1992). Human rod photoreceptor cGMP-gated channel: amino acid sequence, gene structure, and functional expression. J. Neurosci..

[6] Pittler S.J., Lee A.K., Altherr M.R., Howard T.A., Seldin M.F., Hurwitz R.L., Wasmuth J.J., Baehr W. (1992). Primary structure and chromosomal localization of human and mouse rod photoreceptor cGMP-gated cation channel. J. Biol. Chem..

[7] Molday R.S., Warren R., Loewen C., Molday L. (1999). Cyclic GMP-gated channel and peripherin/rds-rom-1 complex of rod cells. Novartis Found. Symp..

[8] Katagiri S., Akahori M., Sergeev Y., Yoshitake K., Ikeo K., Furuno M., Hayashi T., Kondo M., Ueno S., Tsunoda K. (2014). Whole exome analysis identifies frequent CNGA1 mutations in Japanese population with autosomal recessive retinitis pigmentosa. PLoS One.

[9] Paloma E., Martinez-Mir A., Garcia-Sandoval B., Ayuso C., Vilageliu L., Gonzalez-Duarte R., Balcells S. (2002). Novel homozygous mutation in the alpha subunit of the rod cGMP gated channel (CNGA1) in two Spanish sibs affected with autosomal recessive retinitis pigmentosa. J. Med. Genet..

[10] Chen X., Zhao K., Sheng X., Li Y., Gao X., Zhang X., Kang X., Pan X., Liu Y., Jiang C. (2013). Targeted sequencing of 179 genes associated with hereditary retinal dystrophies and 10 candidate genes identifies novel and known mutations in patients with various retinal diseases. Invest. Ophthalmol. Vis. Sci..

[11] Hood D.C., Bach M., Brigell M., Keating D., Kondo M., Lyons J.S., Palmowski-Wolfe A.M. (2008). ISCEV guidelines for clinical multifocal electroretinography (2007 edition). Doc. Ophthalmol..

[12] Li R., Li Y., Fang X., Yang H., Wang J., Kristiansen K. (2009). SNP detection for massively parallel whole-genome resequencing. Genome Res.

[13] Li R., Yu C., Li Y., Lam T.W., Yiu S.M., Kristiansen K., Wang J. (2009). SOAP2: an improved ultrafast tool for short read alignment. Bioinformatics.

[14] Liu T., Jin X., Zhang X., Yuan H., Cheng J., Lee J., Zhang B., Zhang M., Wu J., Wang L. (2012). A novel missense SNRNP200 mutation associated with autosomal dominant retinitis pigmentosa in a Chinese family. PLoS One.

[15] O'Sullivan J., Mullaney B.G., Bhaskar S.S., Dickerson J.E., Hall G., O'Grady A., Webster A., Ramsden S.C., Black G.C. (2012). A paradigm shift in the delivery of services for diagnosis of inherited retinal disease. J. Med. Genet..

[16] Fu Q., Wang F., Wang H., Xu F., Zaneveld J.E., Ren H., Keser V., Lopez I., Tuan H.F., Salvo J.S. (2013). Next–generation sequencing-based molecular diagnosis of a chinese patient cohort with autosomal recessive retinitis pigmentosa. Invest. Ophthalmol. Vis. Sci..

[17] Shanks M.E., Downes S.M., Copley R.R., Lise S., Broxholme J., Hudspith K.A., Kwasniewska A., Davies W.I., Hankins M.W., Packham E.R. (2013). Next-generation sequencing (NGS) as a diagnostic tool for retinal degeneration reveals a much higher detection rate in early-onset disease. Eur. J. Hum. Genet..

[18] Corton M., Nishiguchi K.M., Avila-Fernandez A., Nikopoulos K., Riveiro-Alvarez R., Tatu S.D., Ayuso C., Rivolta C. (2013). Exome sequencing of index patients with retinal dystrophies as a tool for molecular diagnosis. PLoS One.

[19] Glockle N., Kohl S., Mohr J., Scheurenbrand T., Sprecher A., Weisschuh N., Bernd A., Rudolph G., Schubach M., Poloschek C. (2014). Panel-based next generation sequencing as a reliable and efficient technique to detect mutations in unselected patients with retinal dystrophies. Eur. J. Hum. Genet..

[20] Tosi J., Davis R.J., Wang N.K., Naumann M., Lin C.S., Tsang S.H. (2011). shRNA knockdown of guanylate cyclase 2e or cyclic nucleotide gated channel alpha 1 increases photoreceptor survival in a cGMP phosphodiesterase mouse model of retinitis pigmentosa. J. Cell. Mol. Med..

[21] Zhang Q., Zulfiqar F., Riazuddin S.A., Xiao X., Ahmad Z., Riazuddin S., Hejtmancik J.F. (2004). Autosomal recessive retinitis pigmentosa in a Pakistani family mapped to CNGA1 with identification of a novel mutation. Mol. Vis..

[22] Moschos M.M., Chatziralli I.P., Verriopoulos G., Triglianos A., Ladas D.S., Brouzas D. (2013). Correlation between optical coherence tomography and multifocal electroretinogram findings with visual acuity in retinitis pigmentosa. Clin. Ophthalmol..

